# G-Quadruplex Forming Oligonucleotides as Anti-HIV Agents

**DOI:** 10.3390/molecules200917511

**Published:** 2015-09-22

**Authors:** Domenica Musumeci, Claudia Riccardi, Daniela Montesarchio

**Affiliations:** Department of Chemical Sciences, University of Napoli Federico II, via Cintia 21, Napoli I-80126, Italy; E-Mails: domenica.musumeci@unina.it (D.M.); claudia.riccardi@unina.it (C.R.)

**Keywords:** G-quadruplex, modified oligonucleotides, anti-HIV agents, aptamers

## Abstract

Though a variety of different non-canonical nucleic acids conformations have been recognized, G-quadruplex structures are probably the structural motifs most commonly found within known oligonucleotide-based aptamers. This could be ascribed to several factors, as their large conformational diversity, marked responsiveness of their folding/unfolding processes to external stimuli, high structural compactness and chemo-enzymatic and thermodynamic stability. A number of G-quadruplex-forming oligonucleotides having relevant *in vitro* anti-HIV activity have been discovered in the last two decades through either SELEX or rational design approaches. Improved aptamers have been obtained by chemical modifications of natural oligonucleotides, as terminal conjugations with large hydrophobic groups, replacement of phosphodiester linkages with phosphorothioate bonds or other surrogates, insertion of base-modified monomers, *etc.* In turn, detailed structural studies have elucidated the peculiar architectures adopted by many G-quadruplex-based aptamers and provided insight into their mechanism of action. An overview of the state-of-the-art knowledge of the relevance of putative G-quadruplex forming sequences within the viral genome and of the most studied G-quadruplex-forming aptamers, selectively targeting HIV proteins, is here presented.

## 1. Introduction

Complete sequencing of the human genome revealed the presence of ~300,000 distinct sites that can potentially form G-quadruplex (G4) structures [1]. In addition to humans, also other mammals, yeasts and prokaryotic cells exhibit putative G-quadruplex forming sequences which could act as regulatory elements in regions proximal to the transcription start sites of protein-coding genes. Even other organisms, such as viruses, have developed analogous potential regulatory mechanisms. For instance, the presence of G4-forming sequences has been observed in virus genomes such as Epstein-Barr Virus (EBV) [2], Papilloma Virus (HPV) [3], Herpes Simplex Virus-1 (HSV-1) [4] as well as Human Immunodeficiency Virus (HIV) [5], suggesting that the folding/unfolding processes of the G4 structures play important roles in the regulation of viral replication, recombination and gene expression [5]. Thus, treatments with small molecules able to specifically bind G4 structures *in vivo* could interfere with the virus life cycle possibly inhibiting its infectivity. Indeed, several research studies on HIV have been recently addressed at identifying selective small-molecule binders for the G4 structures in the viral genome [5,6] (see paragraph 2). Alternatively, specific oligonucleotide-based aptamers (Apts) structured in G4, recognized by relevant domains of HIV proteins, could be potentially used as anti-viral agents, as demonstrated by a number of literature works carried out in the last two decades, here discussed in paragraph 3.

In this review, focused on HIV, a general overview of the potential role of the G4 structures in the viral life cycle is presented, followed by an extensive discussion on the strategies described in the literature to design and identify effective antiviral agents based on various types of G4-forming oligonucleotide (ON) aptamers.

## 2. Role of the G4 Structures in HIV Life Cycle

**Figure 1:**
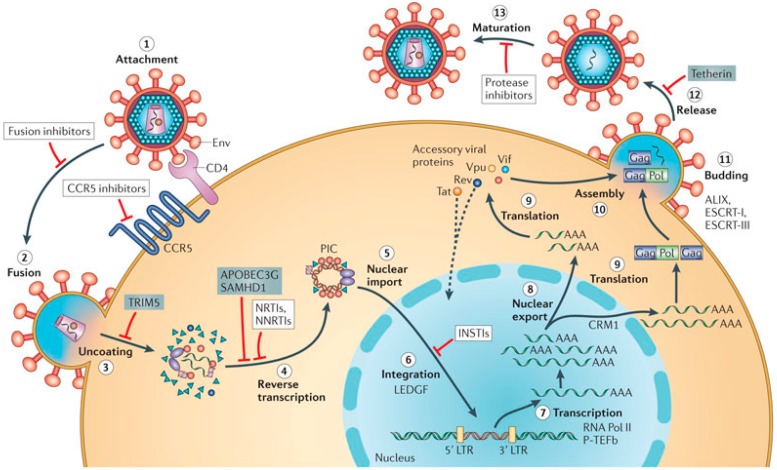
Schematic representation of the replication cycle of HIV (reproduced from Ref. [9] with permission of Nature Publishing Group). The infection begins when the glycoprotein gp120, exposed on the surface of the HIV envelope (Env), recognizes and interacts with the receptor CD4 and the membrane-spanning co-receptor CC-chemokine receptor 5 (CCR5) (step 1), leading to fusion of the viral and cellular membranes and entry of the viral particle into the cell (step 2). Partial core shell uncoating (step 3) facilitates reverse transcription (step 4), which in turn yields the pre-integration complex (PIC). Following import into the cell nucleus (step 5), PIC-associated integrase leads to the formation of the integrated provirus, aided by the host chromatin-binding protein lens epithelium-derived growth factor (LEDGF) (step 6). Proviral transcription (step 7), mediated by host RNA polymerase II (RNA Pol II) and positive transcription elongation factor b (P-TEFb), yields viral mRNAs of different sizes, the larger of which require energy-dependent export to leave the nucleus via host protein CRM1 (Chromosomal Region Maintenance 1 protein, also known as Exportin 1) (step 8). mRNAs serve as templates for protein production (step 9), and genome-length RNA is incorporated into viral particles with protein components (step 10). Viral-particle budding (step 11) and release (step 12) from the cell is mediated by ESCRT (endosomal sorting complex required for transport) complexes and ALIX (ALG-2-interacting protein X) and is accompanied or soon followed by protease-mediated maturation (step 13) to create an infectious viral particle. Each step in the HIV life cycle is a potential target for antiviral intervention; the sites of action of clinical inhibitors (white boxes) and cellular restriction factors (blue boxes) are indicated. INSTI, integrase strand transfer inhibitor; LTR, long terminal repeat; NNRTI, non-nucleoside reverse transcriptase inhibitor; NRTI, nucleoside reverse transcriptase inhibitor.

HIV is an enveloped RNA lentivirus, a subgroup of retroviruses, [7] which attacks the immune system and has been recognized as the causative agent of the acquired immunodeficiency syndrome (AIDS) [8]. After the HIV particle fuses with the host cell surface (Figure 1), the viral particle content is released within the host cell cytoplasm where the viral genome—constituted of two copies of single-stranded, positive-sense RNA, functioning as template—is converted into proviral double-stranded DNA by the viral reverse transcriptase (RT) with the aid of cellular elements (tRNA_Lys3_). The resulting viral DNA is then imported into the nucleus and its insertion into the cellular DNA is catalyzed by the virally encoded integrase (IN). Once integrated, transcription from the viral promoter at the 5′-long terminal repeat (LTR) generates mRNAs that code for several viral proteins and genomic RNA (Figure 1). Alternatively, the provirus may become latent, thus allowing the virus and its host cell to escape detection by the immune system.

**Figure 2:**
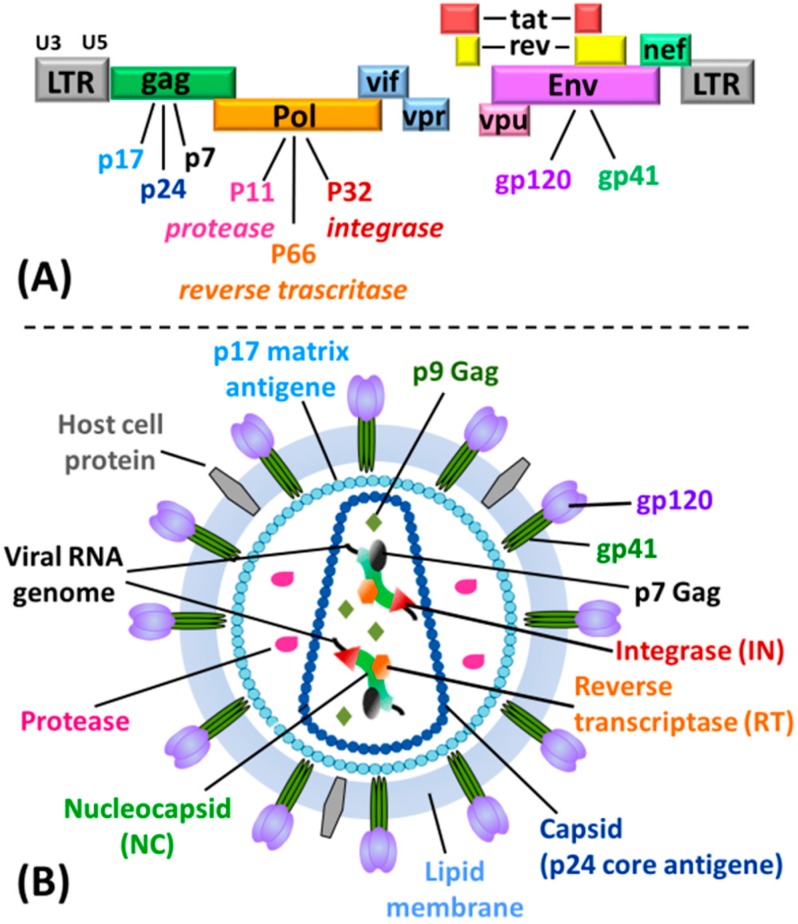
(**A**) HIV genome; (**B**) structure of a HIV virion particle with indication of the potential antiviral targets.

Analysis of the HIV genome highlights the presence of several G-rich regions that can potentially form G4 structures at both RNA and DNA levels, with implications throughout the viral life cycle [5]. The first evidence of G-quadruplex formation in the HIV genome is dated 1993 [10]: a G-rich sequence in the gag region of the HIV genome (Figure 2), near the dimer initiation site (DIS), promotes dimerization of the two viral RNA genome copies forming bi-molecular G4 structures [10,11]. Subsequently, it has been demonstrated that a single-stranded portion of the reverse-transcribed pre-integration HIV genome forms a G-quadruplex structure; this specifically interacts with the viral nucleocapsid protein NCp7 (Figure 2), thereby protecting the pre-integrated genome from nuclease degradation [12]. A recent biophysical study demonstrated molecular chaperone properties for NCp7, which can efficiently promote and stabilize bimolecular G4 formation and is able to anneal G4 structures [13], whereas high concentrations of NCp7 promote G4 unfolding [14]. Taken together, these results reveal that NCp7 can participate in genome recognition, recombination, dimerization and packaging, acting through a mechanism that involves synaptic G4 intermediates [5].

The formation of G4 structures was also demonstrated in the HIV Nef gene (Figure 2) and in the HIV LTR promoter. HIV Nef gene encodes for the corresponding Nef protein, which is a crucial factor for efficient viral replication, infectivity and pathogenesis [15]. On the other hand, in HIV LTR promoter (Figure 2) highly conserved G-rich DNA sequences corresponding to Sp1 and NF-κB binding sites were found to potentially fold into four mutually exclusive G-quadruplex topologies; notably, the equilibrium between these conformations plays a prominent role in regulating promoter activity [16,17]. In the latter cases, G4s act as repressor elements in the transcriptional activation of HIV, representing a good target for antiviral approaches based on G4-stabilizing/inducing agents. In fact, G4-binders showed anti-HIV activity through different mechanisms: either by reducing Nef expression and repressing Nef-dependent enhancement of HIV infectivity [5], or inhibiting the HIV LTR promoter activity [16,17]. A G-quadruplex-forming sequence identical to that of the LTR DNA is present at the 3'-end of the virus RNA genome and can be effectively stabilized by the G4-ligand BRACO-19, thus inhibiting the reverse transcription process at the template level [6,18]. Virological assays demonstrated that BRACO-19 acts at the reverse transcription as well as at the post-integration level during the virus life cycle [6].

## 3. Anti-HIV Active G-Quadruplex-Forming ONs

In the last two decades, many synthetic G-rich oligonucleotides have been identified as promising anti-HIV candidate drugs [5,19,20,21]. Particularly, several G-quadruplex forming aptamers showed ability to act as inhibitors of: (i) virus binding and entry into the target cell; (ii) HIV reverse transcription; or (iii) virus integration (Figure 1), interacting with HIV proteins such as envelope proteins, reverse transcriptase and integrase, and have been developed by either rational design, SELEX (Systematic Evolution of Ligands by EXponential enrichment) or SURF (Synthetic Unrandomization of Randomized Fragments) approaches.

Briefly, SELEX methodology allows to select, from a pool of random-sequence ONs, the molecules that recognize with the highest affinity and specificity a chosen target, such as a protein, a nucleic acid, a small organic compound, or even an entire organism, thanks to the unique three-dimensional foldings adopted by the selected oligonucleotides, able to interact with a specific region of the target [22]. A typical SELEX process starts with the incubation of a random DNA or RNA oligonucleotide library (consisting of about 10^13^–10^15^ different sequence motifs) with the desired target, under conditions suitable for binding. The target-bound oligonucleotide is then partitioned from the unbound and weakly bound sequences, eluted from the target, and finally amplified by PCR (DNA SELEX) or reverse transcription-PCR (RNA SELEX) to give an enriched pool of selected ONs. The latter is used for binding assays with the target in a successive SELEX round, and the process is iterated until the ON pool is reduced to few sequences; then the enriched aptamer is cloned and individually identified by sequencing.

SURF technique involves the synthesis of subsets of oligomers containing a known residue at a fixed position, and equimolar mixtures of different residues at all the other positions. Each subset is then screened in a functional assay and the best subset is identified. A second set of libraries is synthesized and screened, each containing the fixed residue from the previous round, and a second fixed residue. Through successive rounds of screening and synthesis, a single active sequence is finally identified [23].

The rational design approaches are mainly based on the construction of new ON inhibitors with improved properties starting from the sequences selected from combinatorial strategies on a given target, and taking into consideration the information relative to the interaction at molecular level between the ONs and the specific protein-target regions. Alternatively, in the case of HIV, analyzing the viral genome and in particular the sites with putative G4-forming sequences recognizable by important regulatory proteins, the nucleic acid-based aptamers can be selected and optimized with the aid of molecular models built on the basis of the three-dimensional shape of the target protein structures.

### 3.1. Inhibition of Virus Binding and Entry into the Target Cell

The generally poor cellular uptake of oligonucleotides in principle determines high extracellular concentration of G-quadruplex-based candidate drugs. Therefore, independently from the results obtained from *in vitro* studies using different isolated viral protein targets, several G-quadruplex forming ONs are thought to exert their antiviral activity *in vivo* primarily through inhibition of HIV adsorption into host cells and particularly through binding to viral gp120 protein (Figure 1 and Figure 2).

The first G-quadruplex forming oligonucleotide identified as potent anti-HIV agent using the SURF approach was the phosphorothioate 8-mer d(^5′^TTGGGGTT^3′^) (ISIS 5320) [24], which exhibited inhibition of HIV-1 at sub-micromolar concentrations (IC_50_ = 0.3 µM). ISIS 5320 forms a tetramolecular parallel-stranded G-quadruplex, which is able to bind the V3 loop of the envelope glycoprotein gp120 and inhibit virus adsorption and cell fusion (Figure 1) [24,25,26]. The tetrameric G-quadruplex structure provides a rigid and compact complex, which strongly interacts with the cationic V3 loop due to its highly anionic character. A useful modification within this sequence, designed to enhance both thermal and enzymatic stability, has involved the replacement of dG residues with 2′-deoxy, 2′-fluoro-d-arabinofuranosyl nucleic acid (2′F-ANA) monomers, able to stabilize G-tetrads requiring guanines in anti-conformations [27].

Following these studies, Hotoda and coworkers have investigated a large number of G-rich oligonucleotides and promising anti-HIV activity was found in several sequences targeting HIV-1 entry through gp120 binding [28]. In the course of their research, they demonstrated that various G-rich oligomers, bearing suitable substituents at their 5′-end, are non-antisense anti-HIV active compounds. The 6-mer d(^5′^TGGGAG^3′^), successively identified as “Hotoda’s sequence”, was selected as the lead sequence; notably, it resulted to be active against HIV-1 at submicromolar concentrations only when conjugated at the 5′-position with bulky aromatic moieties, essential to produce stabilizing hydrophobic interactions of the aptamer with both the V3 loop as well as the CD4-binding site on viral gp120 [28,29]. Indeed, if the same substituent was inserted at the 3′-end, no activity was observed. It was found that both the G4 structure and the cluster of large aromatic groups at the 5′-ends are crucial for the anti-HIV activity [29]. The most potent *in vitro* antiviral analogue identified was R-95288, bearing the 3,4-dibenzyloxybenzyl (DBB) and 2-hydroxyethylphosphate residues, respectively, at the 5′- and 3′-ends of the d(^5′^TGGGAG^3′^) sequence [28]. Moreover, among the various phosphate-modified analogues investigated, only the oligomer PS7—having one phosphorothioate (P-S) moiety replacing one phosphodiester (P–O) bond in R-95288—was found to have a better pharmacological profile, with higher stability in human plasma and comparable anti-HIV-1 activity [30]. Modification of the guanines within this sequence through N2-methylation led to enhancements both in terms of thermal stability of the resulting G-quadruplex complexes and of *in vitro* anti-HIV activity [31]. In contrast, replacement of dG residues with 8-aza-3-deaza-2′-deoxyguanosine monomers was not beneficial, leading to a general decrease in antiviral activity [32].

In order to better elucidate the structure-activity relationships of G-quadruplex forming oligonucleotides endowed with antiviral activity, the 6-mer d(^5′^TGGGAG^3′^) was chosen as a useful model system in studies carried out by Montesarchio *et al.* [33]. Therein, some representative analogues of the anti-HIV active Hotoda’s sequence—carrying, respectively, the 4,4′-dimethoxytriphenylmethyl (DMT), *tert*-butyldiphenylsilyl (TBDPS) and 3,4-dibenzyloxybenzyl (DBB) groups at the 5′-end—were synthesized and examined in detail by DSC (Differential Scanning Calorimetry), CD (Circular Dichroism) and molecular modeling analyses, in comparison with the unmodified oligonucleotide. The obtained results showed that large aromatic groups at the 5′-end of d(^5′^TGGGAG^3′^) play a crucial role in favoring the G-quadruplex formation processes. The unmodified sequence is indeed able to adopt a tetrameric, parallel stranded G-quadruplex structure, although it is not thermodynamically stable at physiological temperature and its formation is very slow. Conversely, the aromatic groups at the 5′-end of d(^5′^TGGGAG^3′^) dramatically enhance both equilibrium and rate of formation of the G-quadruplex complexes, with *T*_m_ values higher than 70 °C. Interestingly, the overall stability of the investigated G-quadruplexes correlated well with the IC_50_ data; these findings suggested a strict relationship between stability and rate of formation of the G-quadruplexes, on one side, and anti-HIV activity of 5′-modified aptamers, on the other [33].

The successive optimization process of Hotoda’s sequence has therefore been based on the assumption that the kinetically and thermodynamically favored formation of the G-quadruplex complex is a pre-requisite for efficient anti-HIV activity. Several d(^5′^TGGGAG^3′^) derivatives, containing a variety of different large aromatic groups at the 5′-phosphate end, have been synthesized and then subjected to biological evaluation, showing the analogue carrying the (4-benzyloxy) phenylphosphate residue 6-fold more active against HIV-1 than Hotoda’s most active R-95288 (IC_50_ = 0.061 *vs.* 0.37 µM, respectively). All the tested analogues revealed high affinity and specific binding to HIV-1 gp120 and gp41, as determined by Surface Plasmon Resonance (SPR) assays [34].

In a successive variant, a cholesteryl-HEG (hexaethylene glycol) derivative of the Hotoda’s sequence was prepared by a simple and fully automated, on-line phosphoramidite-based solid-phase strategy [35].

A number of novel analogues, bearing different hydrophobic tails at the 5′-ends, have been prepared by Di Fabio *et al.* [36]. The 5′-conjugated oligomers showed pronounced anti-HIV activity, with inhibitory potency in the low micromolar range. Physico-chemical studies indicated that the insertion of lipophilic residues at the 5′-end conferred always improved stability to the resulting G-quadruplex complex (Δ*T*_m_ in the range 20–40 °C). Nevertheless, no direct functional relationship between the thermal stability and anti-HIV activity of the folded conjugated G-quadruplexes would appear in this series of derivatives [36].

The ability to inhibit HIV fusion of a set of Hotoda’s sequence analogues, functionalized with hydrophobic TBDPS groups positioned either at the 5′-hydroxyl group or at 5′-end nucleobases through flexible linkers of different length, was examined through HIV-1 envelop proteins mediated cell-cell fusion assays. In general, high anti-fusion activity was found in almost all the d(^5′^TGGGAG^3′^) analogues investigated, proving that differently positioned TBDPS groups linked at the 5′-end nucleobase favorably affected the binding with HIV envelop proteins. Interestingly, the presence of comparable anti-fusion effects of the Hotoda’s sequences with the TBDPS groups inserted either at the 5′-hydroxyl or 5′-end nucleobase position indicated that a relatively large flexibility in spatial occupation of the bulky group is tolerated, thus offering new opportunities for the design of optimizated analogues of the Hotoda’s sequence. Remarkably, even the 5′-terminal thymine residue—not directly contributing to the G-quadruplex structure—could be replaced by other nucleobases without detectable effects [37].

In an effort to develop novel and effective antivirals, endowed with a more favorable pharmacokinetic profile, a small library of d(^5′^TGGGAG^3′^) derivatives conjugated with mono- or disaccharides (glucose, mannose and sucrose, respectively) at the 3′- or 5′-end was also prepared [38], exploiting a previously optimized fully automated on-line phosphoramidite-based strategy [39,40,41]. Data on the thermal stability of the resulting G-quadruplexes, determined by CD-melting analysis, and on the anti-HIV properties of the novel conjugated oligonucleotides revealed significant bioactivity in those compounds generating the most stable G-quadruplex structures [38].

**Figure 3:**
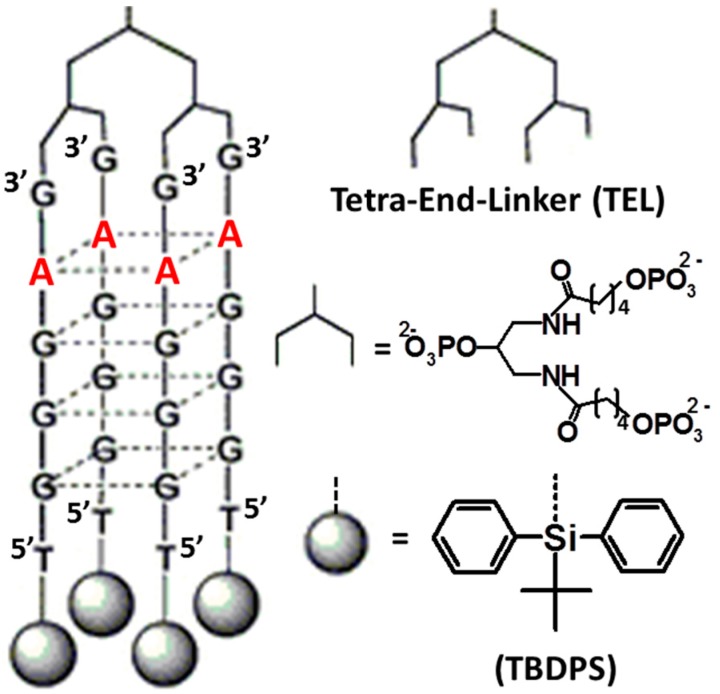
Schematic representation of the best unimolecular G4-forming Tetra-End-linked (TEL)-oligonucleotide carrying the Hotoda’s sequence described in ref. [42].

The concept of favoring the G-quadruplex formation process under physiological conditions through suitable chemical modifications of the oligonucleotide backbone has been examined also from a different perspective, *i.e*., the conversion of the tetrameric G-quadruplex complex generated from d(^5′^TGGGAG^3′^) into a unimolecular or bimolecular, constrained folding. This was realized, in the case of the unimolecular structure, by chemically connecting the 3′- and/or 5′-ends of four d(^5′^TGGGAG^3′^) strands, thus obtaining bunchy oligonucleotides directly built on a solid support, previously functionalized with suitable dendritic spacers [42]. In this way, a small library of anti-HIV aptamers based on a tetra-end-linked (TEL) G-quadruplex structure was realized. The best antiviral candidate resulted to be the compound bearing TBDPS groups at the 5′-end and the longest TEL linker on the bunchy G-rich sequences (Figure 3), showing an EC_50_ (half maximal effective concentration) value of 0.082 µM and an affinity for the HIV-1 gp120 envelope of the same order of magnitude [42]. Successively, starting from this aptamer, depicted in Figure 3, a new mini-library of TEL G-quadruplexes was constructed by substituting the adenine monomers in the second position on the 3’ side of the bunchy ON, with G, C or T monomers, respectively [43]. All the analogues were able to form parallel G4s with high thermal stabilities, analogously to the parent ON; furthermore, they showed comparable binding affinities for HIV-1 gp120 (SPR data) and retained potent anti-HIV activity with EC_50_ in the nM range. The differences in anti-viral activities between the four aptamers [TBDPS-d(^5′^TGGGXG^3′^)]_4_-TEL (with X = A, C, G or T) combined with data of molecular modeling studies, docking the G4s to the V3 loop of gp120, could aid in the understanding of the structural features critical for the biological activity [43].

**Figure 4:**
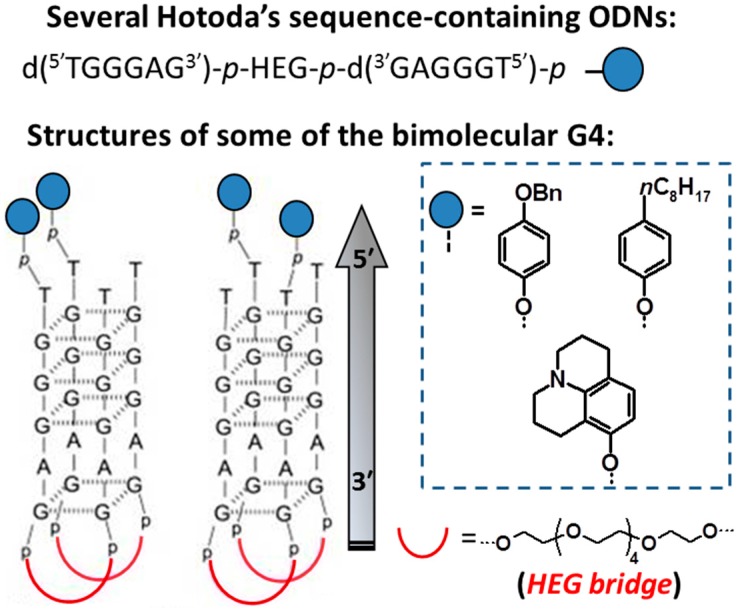
Some anti-HIV-1 ODNs forming bimolecular G4s with a HEG loop connecting two Hotoda's sequence tracts through a 3′-3′ bridge described in ref. [44].

Bimolecular G-quadruplexes, realized by connecting two d(^5′^TGGGAG^3′^) fragments to a HEG loop through 3′-3′ or 5′-5′ bridges, in the first case also possessing aromatic residues conjugated through phosphodiester bonds to the available free ends, were showed to exhibit parallel orientation, high thermal stability, elevated resistance in human serum and high-to-moderate anti-HIV-1 activity with low cytotoxicity (Figure 4) [44]. These molecules also showed significant binding to HIV envelope glycoproteins gp120, gp41 and HSA (Human Serum Albumin), as revealed by SPR assays.

A remarkable increase of the *in vitro* anti-HIV activity of the Hotoda’s sequence has been observed also upon backbone modifications—replacing the natural phosphodiester bond with locked nucleic acid (LNA) residues—and conjugation with (R)-1-*O*-(pyren-1-ylmethyl)glycerol (intercalating nucleic acid, INA) or (R)-1-*O*-[4-(1-pyrenylethynyl)phenylmethyl]glycerol (twisted intercalating nucleic acid, TINA). Incorporation of LNA or INA/TINA monomers produced up to an 8-fold improvement of the anti-HIV-1 activity of this G-rich oligomer; also in this case, the chemically modified sequences were found to form G-quadruplex complexes more thermally stable than the unmodified oligomer [45].

Finally, a structural investigation on the anti-HIV G-quadruplex-forming oligonucleotide d(^5′^TGGGAG^3′^), by using a combined approach including UV, CD, NMR spectroscopy and electrophoretic techniques, was reported by Galeone *et al.* The addition of one thymine at the 3′-end of the Hotoda’s sequence allowed to obtain a single G-quadruplex structure, in which all the experimental data clearly pointed to the presence of an A-tetrad. On this basis, the effects of the incorporation of an 8-methyl-2′-deoxyguanosine at the 5′-end of the oligonucleotide sequence were also investigated [46].

Interesting anti-HIV activity was also found in the sequence d(^5′^GGGTTTTGGG^3′^), forming a dimeric hairpin G-quadruplex (basket-type structure), able to inhibit HIV-1-induced syncytium formation and virus production in peripheral blood mononuclear cells [47]. The antiviral activity of this oligonucleotide increased when the phosphodiester linkages were replaced by phosphorothioate bonds. *In vivo* data showed that the corresponding phosphorothioate sequence is capable of blocking the interaction between gp120 and CD4 (Figure 1), specifically inhibiting the entry of T-cell line-tropic HIV-1 into cells [47].

### 3.2. Inhibition of HIV Reverse Transcription

The HIV reverse transcriptase is a key, multifunctional enzyme with two activities, both cooperating to convert the viral RNA genome into a double-stranded linear DNA in the cytoplasm of the infected cell, *i.e*., a DNA polymerase activity on both RNA and DNA templates, and an RNase-H activity on RNA-DNA hybrid templates, specifically cleaving the RNA strand in a heteroduplex [48,49].

RT is a primary target for HIV inhibition. Anti-HIV drugs currently used in antiviral therapies include the nucleoside RT inhibitors (NRTIs, primarily chain terminators, e.g., AZT, 3TC, ddI, ddC, d4T) and the non-nucleoside RT inhibitors (NNRTIs, non-competitive allosteric inhibitors of polymerization by RT, e.g., snevirapine, delavirdine, efavirenz) (Figure 1). Nucleic acid-based aptamers are included in a third class of RT inhibitors, and some of them contain G-quadruplex structural motifs.

**Figure 5:**
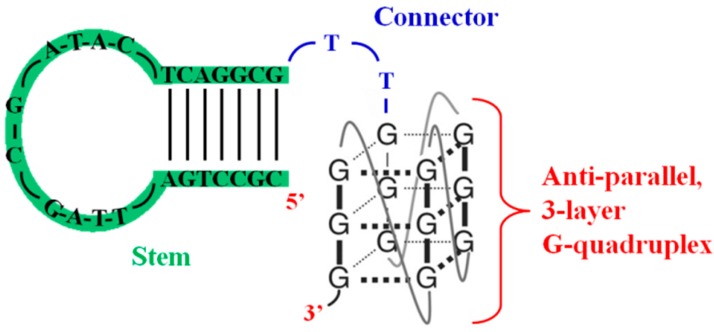
Model structure and sequence of RT6 aptamer described in ref. [50].

A first set of selected G4-based aptamers showed to inhibit the RNA-dependent DNA polymerase activity of HIV-1 RT. In particular, Burke and coworkers [50], while reinvestigating several ssDNA aptamers—previously identified through SELEX as HIV-1 RT selective ligands, with dissociation constant values of approximately 1 nM [51]—recognized, among three of them (RT5, RT6 and RT47), a bimodular structure comprising a 5′-stem-loop module (helical element) connected to a 3′-G-quadruplex module (Figure 5). The authors defined the sequence components required to achieve the functional structures by monitoring RT inhibition for a collection of 60 variants of RT6 and demonstrated that the bimodular structure of the DNA aptamers was essential for RT inhibition. Remarkably, these aptamers were found to inhibit RT from diverse primate lentiviruses with low nM IC_50_ values. 

In order to find inhibitors of the RNase H activity associated with RT, SELEX approaches were developed to isolate DNA aptamers with high affinity for the RNase H domain of the viral transcriptase [52]. Interestingly, these selections led to the identification of several aptamers with G-rich sequences capable of forming G4 structures. Some G4-forming oligodeoxyribonucleotides (ODNs 93 and 112) inhibited the RNase H activity of HIV-1 RT *in vitro* with IC_50_ values in the sub-micromolar range, while no effect was observed on cellular RNase H [52]. Shorter DNA aptamers derived from ODNs 93 and 112, *i.e*., the 16-mer ODNs indicated as 93del and 112del, which maintained the capability to form stable G4 structures—were able to inhibit also HIV-1 integrase in the nanomolar range [53,54]. This dual inhibition can be explained by the structural similarities between the IN active site and RT RNase H domain [55]. Surprisingly, the shortened ODNs were not able to inhibit the RNase H activity to the same extent as the parent, longer ODNs 93 and 112: only 40% and 13% inhibition was observed for 112del and 93del, respectively, both investigated at 6 μM ODN concentration [54].

**Figure 6:**
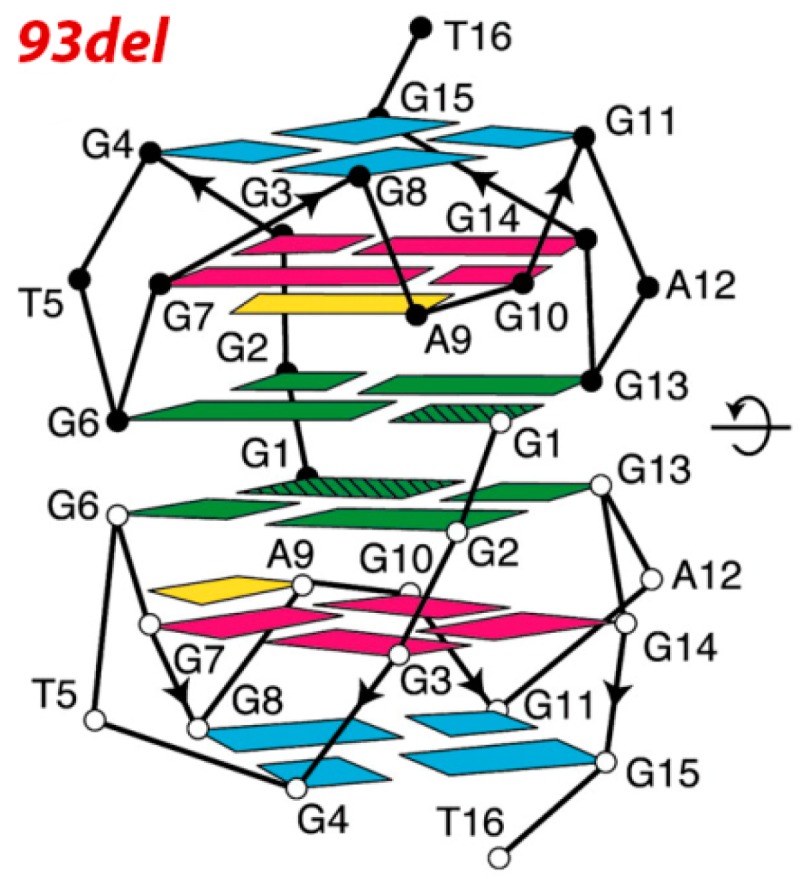
The dimeric G-quadruplex structure of 93del (reproduced from ref. [56] with permission of The National Academy of Sciences, USA—2005).

Detailed NMR investigations showed that, in the presence of K^+^ ions, the 16-mer 93del adopts an unusual dimeric interlocked parallel-stranded G-quadruplex architecture, which is stable even at temperatures higher than 90 °C (Figure 6) [56].

In order to develop highly effective inhibitors of HIV RT, the G4-forming aptamer 93del was recently conjugated to gold nanoparticles (Au NPs) of 13 nm diameter [57]. Generally, aptamers conjugated with NPs (Apt–NPs) are more resistant to nucleases compared with free aptamers [58,59,60,61]. Other striking features of Apt–NPs are their multivalent binding capability and high local aptamer concentration, overall resulting in higher biological activity than free aptamers [62]. Thus, in addition to the DNA sequence, the surface density of the aptamer on the Au NPs and the length of the linker between the aptamer and NPs (typically, a flexible polythymine linker) play important roles in determining the inhibition activity. In a lentivirus infecting experiment, Apt-T_45_–Au NPs showed inhibitory efficiency in the retroviral replication cycle, decreasing by a factor of 40% the viral infectivity [57].

**Figure 7:**
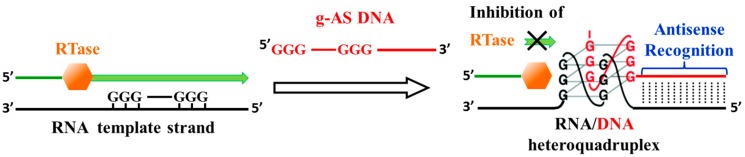
Inhibition of reverse transcription by an antisense-induced RNA-DNA G-quadruplex. A guanine-tethered antisense DNA (g-AS, shown in red) hybridized to a target RNA; then contiguous guanines in both the RNA and the g-AS associated to form a G-quadruplex structure that block the elongation process of RT [63].

Another interesting strategy to inhibit reverse transcription of the viral RNA genome is based on the induced formation of RNA-DNA heteroquadruplex structures on the RNA template, which are able to block the elongation process of HIV RT. These G4 structures on the viral RNA are driven by guanine-tethered antisense (g-AS) ONs consisting of two functionally independent domains: an antisense domain, able to bind a complementary RNA target sequence (adjacent to regions with at least three contiguous guanines), and a contiguous G-run at the 5′-end of the antisense domain, responsible for the assembly into heteroquadruplex structures with the guanine-rich region in target RNA (Figure 7) [63]. Effective inhibition of the RT exclusively depended on the stability of the RNA-DNA heteroquadruplex structures. RT-mediated enzymatic analysis, together with other biophysical analyses, elucidated a cooperative binding of the duplex and G-quadruplex regions in g-AS-RNA complexes.

### 3.3. Inhibition of Virus Genome Integration

Another good target for anti-HIV strategies is the viral enzyme integrase (IN), which is essential for retroviral replication, catalysing the integration of the newly synthesized double-stranded viral DNA genome into the host genomic DNA; importantly, this enzyme has no functional analogues in the host [64,65]. Thus, the inhibition of HIV integrase is of potential clinical relevance in the search for new, efficient and selective antiviral compounds. A remarkable integrase inhibition of HIV-1, with IC_50_ values in the nanomolar range (IC_50_ at ~100 nM), was discovered in the sequence d(^5′^G*TGGTGGGTGGGTGGG*T^3′^), named T30177, a 17-mer composed of only 2′-deoxyguanosines and thymidines, containing single phosphorothioate internucleoside linkages (indicated with *) at its 5′- and 3′-ends. This oligomer showed high nuclease resistance in physiological environments, being capable of folding into a highly stable four-stranded structure [66,67]. T30177, as well as its natural counterpart T30175, tightly binds to HIV-1 integrase, thus blocking the binding of the normal viral DNA substrate to the enzyme [66,67]. T30177 was the first IN inhibitor tested in clinical trials (Zintevir™, developed by Aronex Pharmaceuticals in 1996) [68]. A family of T30177 analogues was also investigated; among these G-rich oligonucleotides, the 16-mer d(^5′^G*GGTGGGTGGGTGGG*T^3′^), known as T30695, proved to be more stable than T30177 and also capable of efficiently inhibiting HIV-1 replication in cell culture [69,70].

To investigate in detail the structure-activity relationships and to further improve inhibition of HIV-integrase activity, a series of analogues of T30695 carrying positively charged residues or large hydrophobic groups were synthesized [71]. In the mini-library of the investigated derivatives, T residues in the loop domains were replaced by 5-amino dU or 5-propynyl dU, and dG residues. From the analysis of the melting temperatures (*T*_m_) of the G-quadruplex structures, and considering the inhibition of integrase activity (IC_50_) and of replication of HIV in cell culture (EC_50_), a relationship between thermal stability of the G-quadruplexes and ability to inhibit viral proliferation in cell cultures was proposed.

A remarkable increase of the *in vitro* anti-HIV activity of T30177 was observed also upon incorporation of LNA or INA/TINA monomers in the natural sequence [45].

**Figure 8:**
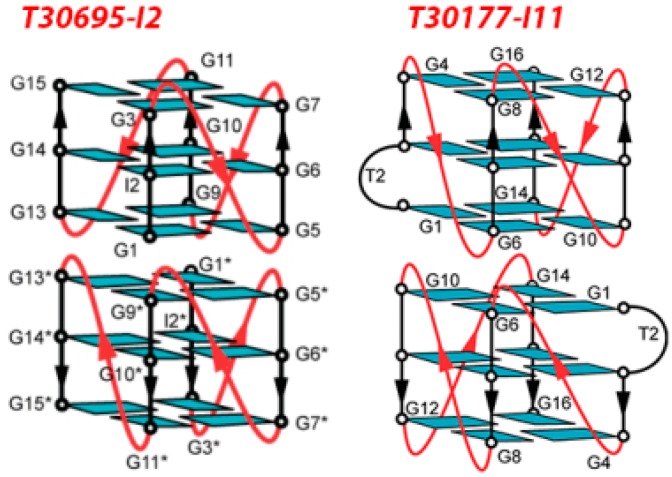
Schematic structures of the dimeric G-quadruplexes adopted by T30695-I2 (**Left**) and T30177-I11 (**Right**) in K^+^ solutions (reproduced respectively from refs. 72 and 73 with permissions of Oxford University Press).

An in-depth NMR study, carried out on T30695 in comparison with a set of extended analogues, has recently demonstrated that this oligomer forms a dimeric structure stabilized by the stacking of two propeller-type parallel-stranded G-quadruplex subunits, in which all the guanine residues participate to the G-tetrad core formation (Figure 8, left) [72]. In parallel, also T30177 has been shown to form a dimeric G-quadruplex structure, with six G-tetrad layers involving the stacking of two propeller-type parallel-stranded G-quadruplex subunits at their 5'-end; all twelve guanines in the sequence participate in G-tetrad formation, with an interruption in the first G-tract due to a thymine forming a bulge between two adjacent G-tetrads (Figure 8, right) [73].

In a recent work [74], describing the identification of high affinity aptamers for the interleukin-6 (IL-6) receptor, one of the selected oligonucleotides, named AID-1, was found to have the same sequence of the already known HIV inhibitor T30923 [d(^5′^GGGT^3′^)_4_]; this is a Zintevir (T30177) analogue, well known for its ability to interfere with HIV infection in cell cultures and inhibit HIV integrase. AID-1 binds IL-6R with a *K*_d_ value in the nanomolar range and does not interfere with IL-6/IL-6R interaction, analogously to all the G-quadruplex-forming aptamers selected in the cited study. Analyzing other HIV inhibitors able to adopt a parallel stranded G-quadruplex structure for the binding to IL-6R, the authors found that also T30175 with an additional thymine nucleotide at position two (compared with AID-1) was able to bind IL-6R with affinity in the nanomolar range. The correlation between IL-6R binding and HIV-integrase inhibition found for the mentioned aptamers remains unclear. Although many HIV integrase-inhibiting oligonucleotides have been described, their mode of action is still not completely understood. Remarkably, HIV-infected cells express higher amounts of IL-6R on their cell surfaces or release increased amounts of soluble IL-6R [75,76]. Considering that IL-6R aptamers can be internalized [77], these HIV inhibitors could display a completely new mechanism of action: indeed, the inhibitors might enter the cell via IL-6R-mediated internalization and target HIV integrase inside the cell.

The inhibitory effect of Zintevir was initially attributed to its inhibition of the 3′ processing activity of the virus integrase [66], but the viral glycoprotein gp120 was later identified as the primary target: Zintevir binding prevented the interaction of gp120 with the CD4 receptor, which is essential for viral entry [78].

Analogously to T30177, also 93del, the unmodified 16-mer of sequence d(^5′^GGGGTGGGAGGAGGGT^3′^) selected for RT inhibition, showed multimodal inhibition of HIV, as mentioned before. As a matter of fact, in addition to reverse transcription, it also *in vitro* inhibits the processing and strand transfer functions of integrase at low nanomolar concentrations, exhibiting a very good selectivity index (>1000) [53,54,55,79]. Additional *in vitro* studies indicated that free 93del and T30923 can efficiently enter human cells, including epithelial (HeLa), hepatic (Huh7) and lymphocytes (H9) cells, with enhanced uptake if in the presence of HIV, acting as a vector [80]. The latter observation opens a valuable opportunity for specific drug delivery to infected cells, which may prevent intracellular side effects from G4 off-targeting.

Using computational methods, including protein-DNA docking and molecular dynamics simulations in explicit solvent, the binding of 93del to HIV integrase was modelled, providing an insight into the interactions of this aptamer with key residues of the catalytic loops of the viral protein, as well as into the molecular mechanism of inhibition, of the utmost importance for the design of new, improved variants of anti-HIV aptamers [81].

Starting from the 93del sequence d(^5′^GGGGTGGGAGGAGGGT^3′^), Phan and Do have recently carried out a rational design to obtain G-rich sequences that form “3+1”-type interlocked dimeric G-quadruplex structures [82]. Three of the newly engineered sequences [s2 = d(^5′^GGGGTGGTGGGTGGGT^3′^), s3 = d(^5′^GGGGTGGGTGGTGGGT^3′^) and s4 = d(^5′^GGGGTGGGTGGGTGGT^3′^)] showed inhibition activity against HIV-1 integrase in the reverse “disintegration” assays, comparable with that of 93del and higher than that of negative controls (a duplex DNA or a G-quadruplex DNA formed by a human telomeric sequence)—even if the sample concentrations used in the assays were in the micromolar range, *i.e*., much higher than the HIV-1 integrase inhibition activity of 93del [82].

## 4. Conclusions

The discovery of G-rich sequences capable of potentially forming G4 structures in regulatory regions of viral genomes, and particularly in HIV, has opened the way to an intense research field, aimed at the identification, on one side, of small molecules which specifically recognize these HIV structures and, on the other side, of efficient G4 aptamers, selectively binding viral proteins, with the aim of inhibiting viral infections.

In this context, it was demonstrated that several G4-forming oligonucleotides can interfere with the virus life cycle, in most cases decreasing infectivity. G4-forming oligomers can be selected as high affinity binders of various viral targets by means of SELEX or rational design approaches, and can be easily chemically modified in order to obtain more active compounds. Therefore, they have great potential as effective and innovative antiviral agents. The most relevant and well characterized targets for HIV are gp120 (involved in virus binding and entry into the target cell), reverse transcriptase and integrase; all these proteins play a vital, specific function in the viral life cycle and, most importantly, have no functional analogues in the host. For each kind of viral protein target, several oligonucleotide sequences with good antiviral activity and satisfactory pharmacological profile have been identified (see Table 1).

With the aim of expanding the repertoire of available oligonucleotide-based antiviral drugs, a large variety of chemical modifications has been introduced into the natural oligonucleotide backbone, thus providing focused libraries of potential candidate drugs obtained either through a combinatorial or a rational design approach. The chemically modified analogues have often showed a better enzymatic or thermodynamic stability, as well as higher inhibitory activity. Remarkably, some oligonucleotide sequences, as 93del and T30177, can exert their activity on more than one viral target, thus providing a multimodal inhibitory approach.

Even though an exceptional wealth of experiments has been devoted to deepen this research field, the discovery of effective anti-HIV candidate drugs is still a big challenge, mainly because of difficulties inherent to *in vivo* studies; therefore, strictly interconnected interdisciplinary competences are more and more needed to finally move drug-like G-quadruplex-forming aptamers into clinic.

**Table 1:** Summary data on the G4-forming oligonucleotides exhibiting anti-HIV activity discussed herein.

ODN Sequence	Structure	HIV Target	Biological Activities
d(^5′^T*T*G*G*G*G*T*T*^3′^) ISIS 5320 [24,25,26]	tetramolecular parallel-stranded G4	V3 loop of gp120	IC_50_ = 0.30 µM
d(^5′^G*G*G*T*T*T*T*G*G*G*^3′^) [47]	bimolecular hairpin G4 (basket-type structure)	HIV-1 gp120	blocks the interaction between gp120 and CD4 inhibiting viral entry
DBB-d(^5′^TGGGAG^3′^)-*p*-OCH_2_CH_2_-OH R-95288 [28,29,30]	tetramolecular parallel-stranded G4	V3 loop and CD4 binding site on gp120	inhibition of the HIV-1IIIB-induced cytopathicity of MT-4 cells (IC_50_ = 0.37 µM)
(4-benzyloxy)phenylphosphate-d(^5′^TGGGAG^3′^) [34]	tetramolecular parallel-stranded G4	HIV-1 gp120 and gp41	IC_50_ = 0.061 µM
[TBDPS-d(^5′^TGGGCG^3′^)]_4_-TEL [43]	unimolecular parallel-stranded G4	HIV-1 gp120	IC_50_ = 0.039 µM
(4-benzyloxy)phenylphosphate-d(^5′^TGGGAG^3′^)-*p*-HEG-*p*-d(^3′^GAGGGT^5′^) [44]	bimolecular parallel-stranded G4	HIV-1 gp120 and gp41	EC_50_ = 0.96 µM
RT6 [50,51]	bimodular structure comprising a 5′-stem-loop element connected to a 3′-G4 module	reverse transcriptase	inhibition RNA-dependent DNA polymerase activity of HIV-1 RT with low nM IC_50_
d(^5′^GG-GGGT-GGGA-GGAG-GGT-AGGCCTTAGGTTTCTGA^3′^) ODN 93 [52]	n.d.	reverse transcriptase	inhibition of RNase H and polymerase activities of the HIV-1 RT: IC_50_ = 0.5 µM; inhibition of viral infectivity :IC_50_ of ~30 nM
d(^5′^CCAGTGGC-GGGT-GGGT-GGGT-GGT-GGGGGGACTTGG^3′^) ODN 112 [52]	n.d.	reverse transcriptase	inhibition of RNase H and polymerase activities of the HIV-1 RT: IC_50_ = 0.5 µM; inhibition of viral infectivity :IC_50_ of ~30 nM
d(^5′^G GGGT-GGGA-GGAG-GGT^3′^) 93del [53,54,55,56]	interlocked dimeric parallel-stranded G4	reverse transcriptase and integrase	inhibition of HIV-1 IN: IC_50_ = 42 nM; inhibition of viral infectivity :IC_50_ of ~20 nM; inhibition of cell fusion in cell at 1 µM
d(^5′^C-GGGT-GGGT-GGGT-GGT^3′^) 112del [53,54]	n.d.	reverse transcriptase and integrase	inhibition of HIV-1 IN: IC_50_ = 9 nM; inhibition of viral infectivity: IC_50_ of ~20 nM
d(^5′^G*TGGTGGGTGGGTGGG*T^3′^)T30177 (Zintevir™) [66,67,68]	n.d.	integrase [66] gp120 [78]	binds to HIV-1 integrase blocking the binding of the normal viral DNA substrate to the enzyme (EC_50_ at ~100 nM) [66]; prevents the interaction of HIV gp120 with the CD4 receptor
d(^5′^G*GGTGGGTGGGTGGG*T^3′^)T30695 [69,70]	n.d.	integrase	inhibition of integrase activities with IC_50_ < 100 nM
[d(^5′^GGGT^3′^)_4_] T30923/AID-1 [71]	n.d.	integrase [66,67] IL-6 receptor [74]	inhibition of integrase activities; binds IL-6R with a *K*_d_ value in the nanomolar range (IL-6*R*-mediated internalization?);
d(^5′^GTGGTGGGTGGGTGGGT^3′^)T30175 [74,75,76]	n.d.	integrase [66,67] IL-6 receptor [74]	inhibition of integrase activities; binds IL-6R with a *K*_d_ value in the nanomolar range (IL-6*R*-mediated internalization?)

n.d. = not determined; DBB = 3,4-dibenzyloxybenzyl; TBDPS = *tert*-butyldiphenylsilyl; TEL = tetra-end-linker; HEG = hexaethylene glycol; *p* = phosphodiester bond; * = phosphorothioate bond.

## References

[1] Huppert J.L., Balasubramanian S. (2005). Prevalence of quadruplexes in the human genome. Nucleic Acids Res..

[2] Murat P., Zhong J., Lekieffre L., Cowieson N.P., Clancy J.L., Preiss T., Balasubramanian S., Khanna R., Tellam J. (2014). G-quadruplexes regulate Epstein-Barr virus-encoded nuclear antigen 1 mRNA translation. Nat. Chem. Biol..

[3] Tlučková K., Marušič M., Tóthová P., Bauer L., Šket P., Plavec J., Viglasky V. (2013). Human papillomavirus G-quadruplexes. Biochemistry.

[4] Artusi S., Nadai M., Perrone R., Biasolo M.A., Palù G., Flamand L., Calistri A., Richter S.N. (2015). The Herpes Simplex Virus-1 genome contains multiple clusters of repeated G-quadruplex: Implications for the antiviral activity of a G-quadruplex ligand. Antivir. Res..

[5] Métifiot M., Amrane S., Litvak S., Andreola M.L. (2014). G-quadruplexes in viruses: Function and potential therapeutic applications. Nucleic Acids Res..

[6] Perrone R., Butovskaya E., Daelemans D., Palù G., Pannecouque C., Richter S.N. (2014). Anti-HIV-1 activity of the G-quadruplex ligand BRACO-19. J. Antimicrob. Chemother..

[7] Mushahwar I.K. (2007). Human Immunodeficiency Viruses: Molecular virology, pathogenesis, diagnosis and treatment. Perspect. Med. Virol..

[8] Barre-Sinoussi F., Chermann J.C., Rey F., Nugeyre M.T., Chamaret S., Gruest J., Dauguet C., Axler-Blin C., Vezinet-Brun F., Rouzioux C. (1983). Isolation of a T-lymphotropic retrovirus from a patient at risk for acquired immune deficiency syndrome (AIDS). Science.

[9] Engelman A., Cherepanov P. (2012). The structural biology of HIV-1: Mechanistic and therapeutic insights. Nat. Rev. Microbiol..

[10] Sundquist W.I., Heaphy S. (1993). Evidence for interstrand quadruplex formation in the dimerization of human immunodeficiency virus 1 genomic RNA. Proc. Natl. Acad. Sci. USA.

[11] Marquet R., Paillart J.C., Skripkin E., Ehresmann C., Ehresmann B. (1994). Dimerization of human immunodeficiency virus type 1 RNA involves sequences located upstream of the splice donor site. Nucleic Acids Res..

[12] Lyonnais S., Gorelick R.J., Mergny J.L., le Cam E., Mirambeau G. (2003). G-quartets direct assembly of HIV-1 nucleocapsid protein along single-stranded DNA. Nucleic Acids Res..

[13] Rajendran A., Endo M., Hidaka K., Tran P.L., Mergny J.L., Gorelick R.J., Sugiyama H. (2013). HIV-1 nucleocapsid proteins as molecular chaperones for tetramolecular antiparallel G-quadruplex formation. J. Am. Chem. Soc..

[14] Kankia B.I., Barany G., Musier-Forsyth K. (2005). Unfolding of DNA quadruplexes induced by HIV-1 nucleocapsid protein. Nucleic Acids Res..

[15] Perrone R., Nadai M., Poe J.A., Frasson I., Palumbo M., Palù G., Smithgall T.E., Richter S.N. (2013). Formation of a unique cluster of G-quadruplex structures in the HIV-1 Nef coding region: Implications for antiviral activity. PLoS ONE.

[16] Perrone R., Nadai M., Frasson I., Poe J.A., Butovskaya E., Smithgall T.E., Palumbo M., Palu G., Richter S.N. (2013). A dynamic G-quadruplex region regulates the HIV-1 long terminal repeat promoter. J. Med. Chem..

[17] Amrane S., Kerkour A., Bedrat A., Vialet B., Andreola M.L., Mergny J.L. (2014). Topology of a DNA G-quadruplex structure formed in the HIV-1 promoter: A potential target for anti-HIV drug development. J. Am. Chem. Soc..

[18] Piekna-Przybylska D., Sullivan M.A., Sharma G., Bambara R.A. (2014). U3 Region in the HIV-1 genome adopts a G-quadruplex structure in its RNA and DNA sequence. Biochemistry.

[19] Shum K.T., Zhou J., Rossi J.J. (2013). Aptamer-based therapeutics: New approaches to combat human viral diseases. Pharmaceuticals.

[20] Jing N. (2000). Developing G-quartet oligonucleotides as novel anti-HIV agents: Focus on anti-HIV drug design. Expert Opin. Investig. Drugs.

[21] Held D.M., Kissel J.D., Patterson J.T., Nickens D.G., Burke D.H. (2006). HIV-1 inactivation by nucleic acid aptamers. Front. Biosci..

[22] Stoltenburg R., Reinemann C., Strehlitz B. (2007). SELEX—A (r)evolutionary method to generate high-affinity nucleic acid ligands. Biomol. Eng..

[23] Ecker D.J., Vickers T.A., Hanecak R., Driver V., Anderson K. (1993). Rational screening of oligonucleotide combinatorial libraries for drug discovery. Nucleic Acids Res..

[24] Wyatt J.R., Vickers T.A., Roberson J.L., Buckheit R.W., Klimkait T., DeBaets E., Davis P.W., Rayner B., Imbach J.L., Ecker D.J. (1994). Combinatorially selected guanosine-quartet structure is a potent inhibitor of human immunodeficiency virus envelope-mediated cell fusion. Proc. Natl. Acad. Sci. USA.

[25] Buckheit R.W., Roberson J.L., Lackman-Smith C., Wyatt J.R., Vickers T.A., Ecker D.J. (1994). Potent and specific inhibition of HIV envelope-mediated cell fusion and virus binding by G quartet-forming oligonucleotide (ISIS 5320). AIDS Res. Hum. Retrovir..

[26] Stoddart C.A., Rabin L., Hincenbergs M., Moreno M., Linquist-Stepps V., Leeds J.M., Truong L.A., Wyatt J.R., Ecker D.J., McCune J.M. (1998). Inhibition of human immunodeficiency virus type 1 infection in SCID-hu Thy/Liv mice by the G-quartet-forming oligonucleotide, ISIS 5320. Antimicrob. Agents Chemother..

[27] Peng C.G., Damha M.J. (2007). G-quadruplex induced stabilization by 2′-deoxy-2′-fluoro-d-arabinonucleic acids (2′-F-ANA). Nucleic Acids Res..

[28] Koizumi M., Koga R., Hotoda H., Momota K., Ohmine T., Furukawa H., Agatsuma T., Nishigaki T., Abe K., Kosaka T. (1997). Biologically active oligodeoxyribonucleotides-IX. Synthesis and anti-HIV-1 activity of hexadeoxyribonucleotides, TGGGAG, bearing 3′- and 5′-end-modification. Bioorg. Med. Chem..

[29] Hotoda H., Koizumi M., Koga R., Kaneko M., Momota K., Ohmine T., Furukawa H., Agatsuma T., Nishigaki T., Sone J. (1998). Biologically active oligodeoxyribonucleotides. 5. 5′-End-substituted d(TGGGAG) possesses anti-human immunodeficiency virus type 1 activity by forming a G-quadruplex structure. J. Med. Chem..

[30] Koizumi M., Koga R., Hotoda H., Ohmine T., Furukawa H., Agatsuma T., Nishigaki T., Abe K., Kosaka T., Tsutsumi S. (1998). Biologically active oligodeoxyribonucleotides. Part 11: The least phosphate-modification of quadruplex-forming hexadeoxyribonucleotide TGGGAG, bearing 3- and 5-end-modification, with anti-HIV-1 activity. Bioorg. Med. Chem..

[31] Koizumi M., Akahori K., Ohmine T., Tsutsumi S., Sone J., Kosaka T., Kaneko M., Kimura S., Shimada K. (2000). Biologically active oligodeoxyribonucleotides. Part 12: N2-methylation of 2′-deoxyguanosines enhances stability of parallel G-quadruplex and anti-HIV-1 activity. Bioorg. Med. Chem. Lett..

[32] Jaksa S., Kralj B., Pannecouque C., Balzarini J., de Clercq E., Kobe J. (2004). How a modification (8-aza-3-deaza-2′-deoxyguanosine) influences the quadruplex structure of Hotoda’s 6-mer TGGGAG with 5′- and 3′-end modifications. Nucleosides Nucleotides Nucleic Acids.

[33] D’Onofrio J., Petraccone L., Martino L., di Fabio G., de Napoli L., Giancola C., Montesarchio D. (2007). 5′-Modified G-quadruplex forming oligonucleotides endowed with anti-HIV activity: Synthesis and biophysical properties. Bioconjugate Chem..

[34] Di Fabio G., D’Onofrio J., Chiapparelli M., Hoorelbeke B., Montesarchio D., Balzarini J., de Napoli L. (2011). Discovery of novel anti-HIV active G-quadruplex-forming oligonucleotides. Chem. Commun..

[35] Musumeci D., Montesarchio D. (2012). Synthesis of a cholesteryl-HEG phosphoramidite derivative and its application to lipid-conjugates of the anti-HIV ^5'^TGGGAG³' Hotoda’s sequence. Molecules.

[36] Romanucci V., Milardi D., Campagna T., Gaglione M., Messere A., D’Urso A., Crisafi E., la Rosa C., Zarrelli A., Balzarini J. (2014). Synthesis, biophysical characterization and anti-HIV activity of d(TG_3_AG) quadruplexes bearing hydrophobic tails at the 5′-end. Bioorg. Med. Chem..

[37] Chen W., Xu L., Cai L., Zheng B., Wang K., He J., Liu K. (2011). d(TGGGAG) with 5′-nucleobase-attached large hydrophobic groups as potent inhibitors for HIV-1 envelop proteins mediated cell-cell fusion. Bioorg. Med. Chem. Lett..

[38] D’Onofrio J., Petraccone L., Martino L., di Fabio G., Iadonisi A., Balzarini J., Giancola C., Montesarchio D. (2008). Synthesis, biophysical characterization, and anti-HIV activity of glyco-conjugated G-quadruplex-forming oligonucleotides. Bioconjug. Chem..

[39] Adinolfi M., de Napoli L., Di Fabio G., Iadonisi A., Montesarchio D., Piccialli G. (2002). Solid phase synthesis of oligonucleotides tethered to oligo-glucose phosphate tails. Tetrahedron.

[40] D’Onofrio J., de Champdoré M., de Napoli L., Montesarchio D., di Fabio G. (2005). Glycomimetics as decorating motifs for oligonucleotides: Solid phase synthesis, stability and hybridization properties of carbopeptoid-oligonucleotide conjugates. Bioconjugate Chem..

[41] Adinolfi M., de Napoli L., di Fabio G., Iadonisi A., Montesarchio D. (2004). Modulating the activity of oligonucleotides: Solid phase synthesis of sucrose-oligonucleotide hybrids. Org. Biomol. Chem..

[42] Oliviero G., Amato J., Borbone N., D’Errico S., Galeone A., Mayol L., Haider S., Olubiyi O., Hoorelbeke B., Balzarini J., Piccialli G. (2010). Tetra-end-linked oligonucleotides forming DNA G-quadruplexes: A new class of aptamers showing anti-HIV activity. Chem. Commun..

[43] D’Atri V., Oliviero G., Amato J., Borbone N., D’Errico S., Mayol L., Piccialli V., Haider S., Hoorelbeke B., Balzarini J., Piccialli G. (2012). New anti-HIV aptamers based on tetra-end-linked DNA G-quadruplexes: Effect of the base sequence on anti-HIV activity. Chem Commun.

[44] Romanucci V., Gaglione M., Messere A., Potenza N., Zarrelli A., Noppen S., Liekens S., Balzarini J., di Fabio G. (2015). Hairpin oligonucleotides forming G-quadruplexes: New aptamers with anti-HIV activity. Eur. J. Med. Chem..

[45] Pedersen E.B., Nielsen J.T., Nielsen C., Filichev V.V. (2011). Enhanced anti-HIV-1 activity of G-quadruplexes comprising locked nucleic acids and intercalating nucleic acids. Nucleic Acids Res..

[46] Virgilio A., Esposito V., Citarella G., Mayol L., Galeone A. (2012). Structural investigations on the anti-HIV G-quadruplex-forming oligonucleotide TGGGAG and its analogues: Evidence for the presence of an A-tetrad. ChemBioChem.

[47] Suzuki J., Miyano-Kurosaki N., Kuwasaki T., Takeuchi H., Kawai G., Takaku H. (2002). Inhibition of human immunodeficiency virus type 1 activity *in vitro* by a new self-stabilized oligonucleotide with guanosine-thymidine quadruplex motifs. J. Virol..

[48] Liu S., Abbondanzieri E.A., Rausch J.W., le Grice S.F.J., Zhuang X. (2008). Slide into action: Dynamic shuttling of HIV reverse transcriptase on nucleic acid substrates. Science.

[49] Abbondanzieri E.A., Bokinsky G., Rausch J.W., Zhang J.X., le Grice S.F.J., Zhuang X. (2008). Dynamic binding orientations direct activity of HIV reverse transcriptase. Nature.

[50] Michalowski D., Chitima-Matsiga R., Held D.M., Burke D.H. (2008). Novel bimodular DNA aptamers with guanosine quadruplexes inhibit phylogenetically diverse HIV-1 reverse transcriptases. Nucleic Acids Res..

[51] Schneider D.J., Feigon J., Hostomsky Z., Gold L. (1995). High-affinity ssDNA inhibitors of the reverse transcriptase of type 1 human immunodeficiency virus. Biochemistry.

[52] Andreola M.L., Pileur F., Calmels C., Ventura M., Tarrago-Litvak L., Toulme J.J., Litvak S. (2001). DNA aptamers selected against the HIV-1 RNase H display *in vitro* antiviral activity. Biochemistry.

[53] De Soultrait V.R., Lozach P.Y., Altmeyer R., Tarrago-Litvak L., Litvak S., Andreola M.L. (2002). DNA aptamers derived from HIV-1 RNase H inhibitors are strong anti-integrase agents. J. Mol. Biol..

[54] Metifiot M., Leon O., Tarrago-Litvak L., Litvak S., Andreola M.L. (2005). Targeting HIV-1 integrase with aptamers selected against the purified RNase H domain of HIV-1 RT. Biochimie.

[55] Andreola M.L. (2004). Closely related antiretroviral agents as inhibitors of two HIV-1 enzymes, ribonuclease H and integrase: “Killing two birds with one stone”. Curr. Pharm. Des..

[56] Phan A.T., Kuryavyi V., Ma J.B., Faure A., Andreola M.L., Patel D.J. (2005). An interlocked dimeric parallel-stranded DNA quadruplex: A potent inhibitor of HIV-1 integrase. Proc. Natl. Acad. Sci. USA.

[57] Shiang Y.C., Ou C.M., Chen S.J., Ou T.Y., Lin H.J., Huang C.C., Chang H.T. (2013). Highly efficient inhibition of human immunodeficiency virus type 1 reverse transcriptase by aptamers functionalized gold nanoparticles. Nanoscale.

[58] He X.X., Wang K., Tan W., Liu B., Lin X., He C., Li D., Huang S., Li J. (2003). Bioconjugated nanoparticles for DNA protection from cleavage. J. Am. Chem. Soc..

[59] Seferos D.S., Prigodich A.E., Giljohann D.A., Patel P.C., Mirkin C.A. (2009). Polyvalent DNA nanoparticle conjugates stabilize nucleic acids. Nano Lett..

[60] Milano G., Musumeci D., Gaglione M., Messere A. (2010). An alternative strategy to synthesize PNA and DNA magnetic conjugates forming nanoparticle assembly based on PNA/DNA duplexes. Mol. BioSyst..

[61] Musumeci D., Oliviero G., Roviello G.N., Bucci E.M., Piccialli G. (2012). G-quadruplex-forming oligonucleotide conjugated to magnetic nanoparticles: Synthesis, characterization, and enzymatic stability assays. Bioconjugate Chem..

[62] Musumeci D., Montesarchio D. (2012). Polyvalent nucleic acid aptamers and modulation of their activity: A focus on the thrombin binding aptamer. Pharmacol. Ther..

[63] Hagihara M., Yamauchi L., Seo A., Yoneda K., Senda M., Nakatani K. (2010). Antisense-induced guanine quadruplexes inhibit reverse transcription by HIV-1 reverse transcriptase. J. Am. Chem. Soc..

[64] Lutzke R.A., Plasterk R.H. (1997). HIV integrase: A target for drug discovery. Genes Funct..

[65] Chiu T.K., Davies D.R. (2004). Structure and function of HIV-1 integrase. Curr. Top Med. Chem..

[66] Ojwang J.O., Buckheit R.W., Pommier Y., Mazumder A., de Vreese K., Esté J.A., Reymen D., Pallansch L.A., Lackman-Smith C., Wallace T.L. (1995). T30177, an oligonucleotide stabilized by an intramolecular guanosine octet, is a potent inhibitor of laboratory strains and clinical isolates of human immunodeficiency virus type 1. Antimicrob. Agents Chemother..

[67] Mazumder A., Neamati N., Ojwang J.O., Sunder S., Rando R.F., Pommier Y. (1996). Inhibition of the human immunodeficiency virus type 1 integrase by guanosine quartet structures. Biochemistry.

[68] Marchand C., Maddali K., Metifiot M., Pommier Y. (2009). HIV-1 IN inhibitors: 2010 Update and perspectives. Curr. Top. Med. Chem..

[69] Jing N., Rando R.F., Pommier Y., Hogan M.E. (1997). Ion selective folding of loop domains in a potent anti-HIV oligonucleotide. Biochemistry.

[70] Jing N., Hogan M.E. (1998). Structure-activity of tetrad-forming oligonucleotides as a potent anti-HIV therapeutic drug. J. Biol. Chem..

[71] Jing N., de Clercq E., Rando R.F., Pallansch L., Lackman-Smith C., Lee S., Hogan M.E. (2000). Stability-activity relationships of a family of G-tetrad forming oligonucleotides as potent HIV inhibitors. A basis for anti-HIV drug design. J. Biol. Chem..

[72] Do N.Q., Lim K.W., Teo M.H., Heddi B., Phan A.T. (2011). Stacking of G-quadruplexes: NMR structure of a G-rich oligonucleotide with potential anti-HIV and anticancer activity. Nucleic Acids Res..

[73] Mukundan V.T., Do N.Q., Phan A.T. (2011). HIV-1 integrase inhibitor T30177 forms a stacked dimeric G-quadruplex structure containing bulges. Nucleic Acids Res..

[74] Magbanua E., Zivkovic T., Hansen B., Beschorner N., Meyer C., Lorenzen I., Grötzinger J., Hauber J., Torda A.E., Mayer G. (2013). d(GGGT)_4_ and r(GGGU)_4_ are both HIV-1 inhibitors and interleukin-6 receptor aptamers. RNA Biol..

[75] Honda M., Yamamoto S., Cheng M., Yasukawa K., Suzuki H., Saito T., Osugi Y., Tokunaga T., Kishimoto T. (1992). Human soluble IL-6 receptor: Its detection and enhanced release by HIV infection. J. Immunol..

[76] Novick D., Shulman L.M., Chen L., Revel M. (1992). Enhancement of interleukin 6 cytostatic effect on human breast carcinoma cells by soluble IL-6 receptor from urine and reversion by monoclonal antibody. Cytokine.

[77] Zhou J., Rossi J.J. (2011). Cell-specific aptamer-mediated targeted drug delivery. Oligonucleotides.

[78] Este J.A., Cabrera C., Schols D., Cherepanov P., Gutierrez A., Witvrouw M., Pannecouque C., Debyser Z., Rando R.F., Clotet B. (1998). Human immunodeficiency virus glycoprotein gp120 as the primary target for the antiviral action of AR177 (Zintevir). Mol. Pharmacol..

[79] Faure-Perraud A., Métifiot M., Reigadas S., Recordon-Pinson P., Parissi V., Ventura M., Andreola M.L. (2011). The guanine-quadruplex aptamer 93del inhibits HIV-1 replication *ex vivo* by interfering with viral entry, reverse transcription and integration. Antivir. Ther..

[80] Métifiot M., Faure A., Guyonnet-Duperat V., Bellecave P., Litvak S., Ventura M., Andréola M.L. (2007). Cellular uptake of ODNs in HIV-1 human-infected cells: A role for viral particles in DNA delivery. Oligonucleotides.

[81] Sgobba M., Olubiyi O., Ke S., Haider S. (2012). Molecular dynamics of HIV1-integrase in complex with 93del—A structural perspective on the mechanism of inhibition. J. Biomol. Struct. Dyn..

[82] Phan A.T., Do N.Q. (2013). Engineering of interlocked DNA G-quadruplexes as a robust scaffold. Nucleic Acids Res..

